# Structural Behavior of FRP-Retrofitted RC Beams under Combined Torsion and Bending

**DOI:** 10.3390/ma15093213

**Published:** 2022-04-29

**Authors:** Ahad Amini Pishro, Shiquan Zhang, Zhengrui Zhang, Yana Zhao, Mojdeh Amini Pishro, Lili Zhang, Qihong Yang, Victor Postel

**Affiliations:** 1 Civil Engineering Department, Sichuan University of Science and Engineering, Zigong 643000, China; zhangzhengrui@suse.edu.cn (Z.Z.); zhao_yana@suse.edu.cn (Y.Z.); zhanglilifei@163.com (L.Z.); 2 School of Mathematics, Sichuan University, Chengdu 610065, China; shiquanzhang@scu.edu.cn; 3 School of Architecture and Design, Southwest Jiaotong University, Chengdu 610031, China; aminimojdeh95@yahoo.com; 4 Laboratoire Energétique Mécanique Electromagnétisme, UPL, Université Paris Nanterre, 50 rue de Sèvres, 92410 Ville d’Avray, France; victor.postel@hotmail.com

**Keywords:** FRP, retrofitted RC beams, ABAQUS, ANN, combined loading

## Abstract

Fiber-reinforced polymers (FRPs) retrofit reinforced concrete (RC) structures. ABAQUS finite element software was used to perform numerical parametric analysis on a group of RC beams in this research. All specimens were retrofitted by FRP strips as an external retrofitting and experimentally tested up to previous researchers’ failure points. The range of subjects examined in these RC beams included cracking torque, ultimate torque, angle of twist, and the effect of using FRP on these subjects. We applied artificial neural networks (ANNs) to predict the structural behavior of RC beams under combined torsion and bending to develop the research accuracy. After testing, the ANN results were compared with the ABAQUS results. Consequently, a reasonable examination of the determined mathematical and trial results confirmed this study’s logical accuracy in predicting retrofitted RC beams’ structural behavior under combined loading.

## 1. Introduction

Concrete structures may be damaged and need to be repaired, strengthened, or rehabilitated [1]. This damage is discussed in terms of both the material and structure. Material damage in concrete structures occurs as steel corrosion.

Many countries are located in earthquake-prone areas, and earthquake forces may not have been appropriately considered in the design of many buildings in recent years, so many structures should be retrofitted against earthquake forces [2]. In many cases, changing the use of a structure from residential to commercial or administrative may be of interest [3,4]. Assuming that the structure is adequately designed, and all factors, including possible loads, especially earthquake loads, are taken into account, the new-use structure will be placed under new loadings that were not considered in the preliminary design stage. If the structure is adequately designed, it is still possible that it is not implemented correctly as calculated and designed, leading to several problems such as inappropriate reinforcement, using rebars of the wrong size and number, noncompliance with the length of rebar requiring anchors, failure to implement connections correctly, use of unsuitable cement, use of not enough cement, and inappropriate concrete curing in the implementation phase [5,6,7,8,9]. Therefore, strengthening and repairing concrete structures also arises when changing the use of structures.

In some cases, parts of regulations and load factors may be changed due to new scientific findings. Structures based on previous principles must be consistent with the new regulations [10,11]. In some cases, strengthening and repair of the structure may be needed for this aim.

The fragility of concrete, torsion, and bending failure play undesirable roles in structural failure [12]. Due to the complexity of combined loading mechanisms and with different parameters affecting the strength of retrofitted RC beams, it sounds challenging to establish a comprehensive estimation model of the structural strength of these kinds of members under torsion and bending [13].

Lu et al., provided research on the bond–slip relationship between FRP and concrete [14]. They created a comprehensive database and performed a series of pull tests on FRP-concrete-bonded joints to propose a new bond–slip model. Their study showed that the bond–slip between FRP and concrete is based on the meso-scale FEM prediction and not according to the axial strain of the FRP. Wu and Jiang also provided a parametric and analytical database of 628 experiments to investigate the bond–slip between concrete and external retrofitting FRP sheets. They applied the FRP-concrete width parameter to offer precise models for bond strength and fracture energy [15].

Sri Tudjono et al. [16] researched the influence of FRP confinement on beams exposed to bending and shear. They studied the FRP flexural and u-molded shear retrofitting mix to assess the beam’s capacity increase following ACI 440. Significant commitments concerning the conduct of RC beams under consolidated shear and twist were made by a few specialists [17,18,19,20]. Ameli and Ronagh [21] presented an analytical method for assessing the torsional capacity of FRP-retrofitted RC beams by considering concrete, steel, and FRP interactions. Gopal Charan Behera [22] studied the torsional capacity of RC beams with U-jacketing reinforcements. Their exploration results showed that torsion’s condition impacts the torque twist reaction of a reinforcement “U” wrap beam more than the amount of reinforcement. Eventually, RC beams are more effective in opposing torque than other twist states. A point of achievement in investigating RC beams under joined shear and torsion was the work introduced by both Hsu and Rahal, and Collins [20,23]. Another essential point in RC beams’ set of experiences under consolidated loading was Greene and Belarbi [24]. They showed a “Combined-Action Softened Truss Model”, which depended on the “Softened Truss Model” by Hsu and Mo, for an unadulterated twist with upgrades over existing models [20,25]. Experimental studies, theoretical estimations, and numerical reproductions showed that strengthening the RC beams with externally bonded carbon-fiber-reinforced polymer (CFRP) sheets in the tension zone considerably increased bending and deflections as the crack width increased [26,27,28,29,30]. Many researchers have used externally bonded FRP composites to improve the flexural capacity of RC structures. However, very few studies are available that concentrate on the effect of GFRP sheets on the structural capacity of RC beams under combined torsion and bending [31,32].

FRP composites can be viably utilized as external reinforcements for upgrading structurally insufficient RC structures [33,34,35]. Santhakumar et al. [36] presented numerical research on non-retrofitted and retrofitted RC beams exposed to combined bending and torsion utilizing the finite element method. Woo et al. [37] explored the retrofitting effect of prestressed FRP plates on RC beams and proposed a strength-prediction method. They found that prestressed FRP plates can increase the cracking, yielding, and ultimate load. Moreover, the workableness of the beam was also enhanced.

Akbarzadeh et al. [38] led a trial program to examine the flexural conduct and moment redistribution of reinforced high-strength concrete (RHSC) beams strengthened with CFRP and GFRP sheets. Majid Mohammed Ali Kadhim [39] focused on the behavior of a high-strength concrete continuous beam retrofitted by a CFRP sheet with various lengths. Moreover, as of late, through either experimental, finite element, or analytical methodologies [40,41], the broad examination has been directed to investigate the behavior of reinforced concrete beams retrofitted by externally bonded FRP sheets to enhance their bending and shear performance.

The failure mode of reinforced concrete structural members retrofitted by FRP can be changed, while torsion plays a vital role in loading. A. Ganganagoudar et al. [42] presented an analytical and finite element (FE) study to propose a modified softened membrane model for torsion (SMMT-FRP). Their results proved an increase in structural behavior such as post-cracking stiffness, ultimate strength, and localized damage of RC retrofitted members. Saumitra Jain et al. [43] carried out an experimental study on an emergency retrofitting model of damaged reinforced concrete columns under axial compression loading. Their experimental results of testing six short RC columns showed that compared to Near-Surface Mounting (NSM), CFRP retrofitting and quick-setting cement mortar, the hybrid strengthening technique has a better efficiency in increasing the structural capacities of their specimens. M. Chellapandian et al. [44] conducted a comprehensive study to investigate the effectiveness of hybrid FRP in restoring the structural capacities of damaged plain concrete (PC) and reinforced concrete (RC) columns under cyclic compression loading. Their analytical and finite element analysis results proved the effectiveness of the hybrid retrofitting technique in restoring the stiffness and strength of their column specimens.

Recently, researchers have applied Artificial Neural Networks (ANNs) in structural engineering to predict the engineering parameters and behavior of structures and elements [45,46,47,48,49,50]. The application of ANNs, compared to experimental studies, provides less time- and expense-consuming methods in order to better understand structural concepts. J. Amani et al. [51] applied ANNs to study the shear strength of reinforced concrete beams utilizing an adaptive neuro-fuzzy inference system (ANFIS). Their outcomes showed that the ANN model preferred shear strength prediction over the ANFIS model. Likewise, the ANN and ANFIS models are more exact than the experimental ACI and ICI codes. H. Naderpour et al. [52] conducted research using ANNs to estimate the behavior of RC structural members strengthened with FRP. The predicted behavior of the FRP-confined member showed sensible agreement with the results of experimental programs, which indicated that the neural network is a reliable and robust tool for costly and protracted experimental programs.

The present study aimed to numerically analyze unstrengthened and FRP-retrofitted RC beams under the simultaneous effects of torsion and bending to better understand the behavior of these beams and the strengthening effects of FRP on RC beams. Moreover, an ANN model was used to predict the structural behavior of FRP-confined RC beams. The technical literature review indicates that a few studies have been conducted in this field, and numerical studies are rare. On the other hand, there is no analytical equation for determining the capacity of retrofitted RC beams under combined loading. Considering the points mentioned above, conducting a numerical analysis seems necessary.

The application of Mean Square Error (MSE) and Artificial Neural Network (ANN) to predict the structural behavior of FRP retrofitted beams under combined loading is the novelty of this paper. In this research, we applied the finite element software ABAQUS CAE (Version 6.10.1; Karlsson & Sorensen Hibbitt Inc.: Pawtucket, RI, USA) to calibrate and check the validity of the experimental studies conducted by previous researchers. After providing a precise numerical database, we trained our ANN model to predict the structural responses of FRP-retrofitted RC beams. Finally, a concept of applied mathematics was used to prove the accuracy of this research work.

## 2. Materials and Methods

A total of seven rectangular, reinforced concrete beams retrofitted and non-retrofitted with GFRP under combined torsion and bending were studied by Vishnu et al. [53]. All beams had a 150 mm × 150 mm cross-section and were 1700 mm in length. The beams were bolstered with four- to ten-millimeter diameter steel bars within the longitudinal direction and eight-millimeter diameter stirrups in the crosswise direction, spaced at 150 mm center to center.

Table 1 shows the diverse wrapping configurations used to retrofit rectangular beams [53].

Initially, one concrete beam specimen without any GFRPs, namely, BTCON, was tested to obtain the essential structural response. These rectangular-section beam specimens were supported and loaded up to failure. This test was utilized in the present numerical study to calibrate the material parameters of the concrete material model. This experimental study showed that using GFRP strips to retrofit RC beams enhances the torsional strength. Diagonal strip wrapping is more efficient in resisting the torsional moment than vertical stripes.

Both the experimentally and analytically determined retrofitting material properties are presented in Table 2.

It may be presumed that the reinforcing schemes utilized significantly affected the conduct in terms of strength and ductility. Additionally, the FRP strain yielded either tension or compression in two orthogonal directions, which follows the notable conduct of beams in shear and torsion. The beam creates two arrangements of symmetrical forces, one compression and one tension force. When comparing the FRP sheets and the transverse steel strain, the FRP strain is higher than that of the transverse steel (approximately 50% higher). This contrast is ascribed to how the FRP is applied to the cross-area outer edge further away from the shear center of the section, expanding the twist’s strain. Since FRP is a linear material, the increment in the strain will increase the force resisted by the FRP, and in this manner, give a higher commitment to the strength [54,55,56]. The measured mean concrete strength and a summary of tests for each specimen are listed in Table 3.

### 2.1. Material Modeling

The previous auxiliary test results were utilized herein to define the material response. The GFRP sheets were assumed to be orthotropic under plane stress conditions. The material properties reported in Table 2 were adopted. Moreover, some specimens failing due to FRP rupture, damage, and failure of the FRPs were explicitly modeled by adopting the Hashin damage model [57] incorporated in ABAQUS. Only tensile failure in the principal direction was considered, and the ultimate stress reported in Table 2 was adopted. In contrast, other failure modes were deemed irrelevant to the present study. Fracture energy equal to 0.01 was assumed to simulate damage evolution, which was rapid due to the severe stress concentration following the onset of failure. Longitudinal reinforcing bars and steel stirrups were considered to be elastic–isotropic hardening. The experimentally obtained stress–strain curves for the steel used for these parts were converted into the valid stress-logarithmic plastic-strain format to Equations (1) and (2) and utilized to define the material response.

(1) $$\sigma_{true}=\sigma_{nom}\left(1+\varepsilon_{nom}\right)$$

(2) $$\varepsilon_{ln}^{pl}=\ln\left(1+\varepsilon_{nom}\right)-\frac{\sigma_{true}}{E}$$

The damaged plasticity model for concrete available in the ABAQUS material library was adopted to model the concrete response since it has been shown to perform satisfactorily in similar applications [58]. All material parameters were initially calibrated to accurately simulate the control beam specimens’ response. The Young’s modulus E and tensile strength $f_{ct}$ were determined according to ACI [59] as a function of the compressive cylindrical strength, while Poisson’s ratio was assumed to be equal to 0.2. The resin-rich layer, within which the FRP strip’s debonding from the concrete volume occurs, was modeled as a cohesive zone [60] endowed with a traction-separation response. The quadratic stress-based damage initiation criterion in ABAQUS was adopted, equaling all three principal directions. The stress limit in all three main directions equaled the respective tensile concrete strength. Damage evolution was defined in terms of fracture energy with linear softening. Simultaneously, mixed-mode behavior was accounted for according to the model proposed by Benzeggagh and Kenane [61], with a power coefficient equal to 1.45, following the proposals of Obaidat et al. [62]. The best results were obtained for fracture energy in both shearing and peeling modes equal to 0.25 ($G_{II}=G_{III}=0.25\;N/{\mathrm{mm}}^{2}$), while the fracture energy $G_{I}$ associated with the opening mode was set equal to 0.025. Overall, it should be noted that the overall response is relatively insensitive to the assumed elastic stiffness and limiting stress adopted. These properties significantly affect the numerical cohesive zone length [63,64], which defines the minimum required mesh size.

### 2.2. Nonlinear Analysis and Discretization

Due to severe convergence problems typically associated with a strongly nonlinear response and materials exhibiting a nonmonotonic stress–strain reaction [65], such as concrete and cohesive elements, the explicit dynamics solver ABAQUS/EXPLICIT was employed to perform the nonlinear analyses. Linear 8-noded brick elements were adopted to discretize the concrete beam and loading plates. In contrast, the longitudinal reinforcing bars and the steel stirrups were discretized with linear truss elements embedded in the concrete region (i.e., no relative displacement between the reinforcement and concrete was allowed). Linear 4-noded shell elements were used to discretize the FRP sheets. The degrees of freedom (DOFs) were tied to the respective DOFs of the underlying 8-noded 3D cohesive elements. A uniform mesh size of 25 mm was adopted upon extensive mesh convergence studies for the concrete beam, cohesive zone, and FRP sheets. The mesh size was assigned to minimize computational time while maintaining accuracy. The quasi-static response was achieved by specifying a slow displacement rate and checking that the kinetic energy was smaller than 2% of the internal energy for the most significant part of the analysis.

**Figure 1:**
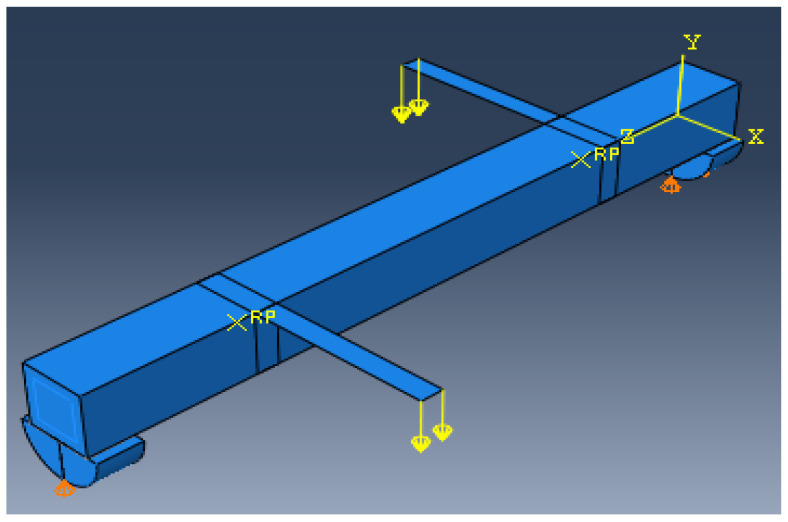
Rectangular beams modeled in ABAQUS.

The mean measured specimen geometries were utilized to simulate the test specimens. The entire 3D geometry of the concrete volume and FRP strips was modeled with 3D brick-type F.E. elements. The FRP interface and the concrete were simulated as a cohesive zone with a suitable traction separation response. Using the Tie option of ABAQUS allows a model of two different surfaces (FRP and Concrete) to have two separate motions from each other. In other words, two separate motions for master and slave surfaces at their interface have been considered. Figure 1 shows the rectangular beams modeled in ABAQUS.

## 3. Results and Discussion

### 3.1. Evaluation of Retrofitted Beams under Combined Torsion and Bending

All beams were analyzed using ABAQUS Finite Element software and eventually compared to the experimental results. The cracking torque, ultimate torque, ultimate angle of twist, and effects of fibers on them for reference, FT, FL, 100s100, CO and 100s100, DS, and CO and DS beams are presented in Table 4 as the experimental results [53]. Additionally, Table 5 shows the numerical analysis outcomes obtained by ABAQUS.

The numerical and experimental results of the cracking torque, ultimate torque, and ultimate angle of twist for the beams studied by Vishnu et al. [53] are relatively consistent. Comparing the numerical and experimental values for these beams in Table 6 shows the right consistency and reliable prediction of RC beam behavior retrofitted with FRPs by FEM software (ABAQUS).

Comparing the results shows a remarkable correspondence between the numerical cracking and ultimate torque and their experimental counterparts. The cracking torque from the reference beam’s numerical study is 1.85% higher than the experimental values. In contrast, numerical predictions for this beam’s ultimate torque are 4% lower than similar values from the experimental study. Furthermore, the ultimate angle of the twist of the reference beam from the numerical analysis is 3.4% lower than the respective experimental values. Using the S4R element to model the fibers in retrofitted beams and assuming orthotropic properties, the samples’ nonlinear numerical analysis results show the right consistency.

Due to improper fiber orientation, the FL beam maintains a lower ultimate torque than the other beams.

CO and DS and FT beams have the highest fiber ratio and orientation and the highest ultimate torque compared to other orientations. The CO and 100S100 beam has the most plasticity, considering fiber orientation. The cracking moment and ultimate torque are highest in the FT beam for different modes in GFRP modeling.

### 3.2. Application of ANN

**Figure 2:**
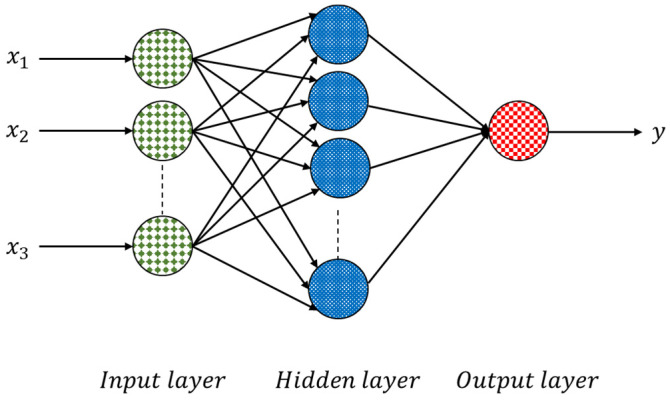
Structure of an ANN neural network.

ANNs have proven to be standard functional approximations that can fit complex functions or solve classification problems. The most typical ANN structure consists of three layers—a labeled input layer, a hidden layer, and an output layer—as shown in Figure 2.

ANN is considered a feed-forward neural network, which means that there is one direction from the input to output neurons. The information processing goes in this direction. Based on the backpropagation algorithm, training algorithms perform learning and error-correction processes related to the input and output data layers. The ANN receives the input data to calculate the error value by assessing the target and output values. ANNs benefit from a group of neurons and their relationships, which differ under their assigned weights. The ANN adjusts the weights of interconnections between neurons to minimize the error. The network keeps this process going to obtain a logical minimum error. As our problem is a regression problem, the most commonly used loss function is the mean square error (MSE), the sum of the prediction data’s squares and the original data’s corresponding point error. The MSE can predefine the logical minimum error.

(3) $$\mathrm{MSE}\left(y,\;y^{\prime }\right)=\frac{\sum_{i=1}^{n}{\left(y_{i}-y_{i}^{\prime }\right)}^{2}}{n}\;$$

**Figure 3:**
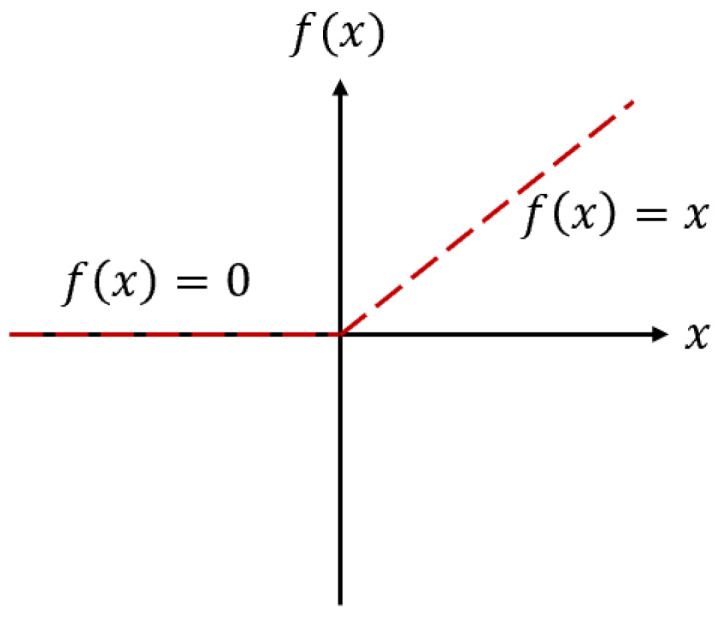
The activation function of neurons (f).

We used the rectified linear unit (ReLU) function as the neurons’ activation function (f), as shown in Figure 3.

(4) $$ReLU\left(x\right)=\{\begin{array}{c} x\;if\;x>0\\ 0\;if\;x\leq 0 \end{array}$$

The following mathematical equations describe a neuron K: In these equations, the output signal of the neuron is presented by $y_{k}$, the activation function is represented by f, the linear output is $u_{k}\;$, the bias term is indicated by $b_{k}$ and input signals and interconnection weights are denoted by $x_{i}$ and $w_{ki}$, respectively.

(5) $$y_{k}=f\left(u_{k}+b_{k}\right),$$

(6) $$u_{k}=\overset{N}{\underset{i=1}{\sum }}w_{ki}x_{i}$$

**Figure 4:**
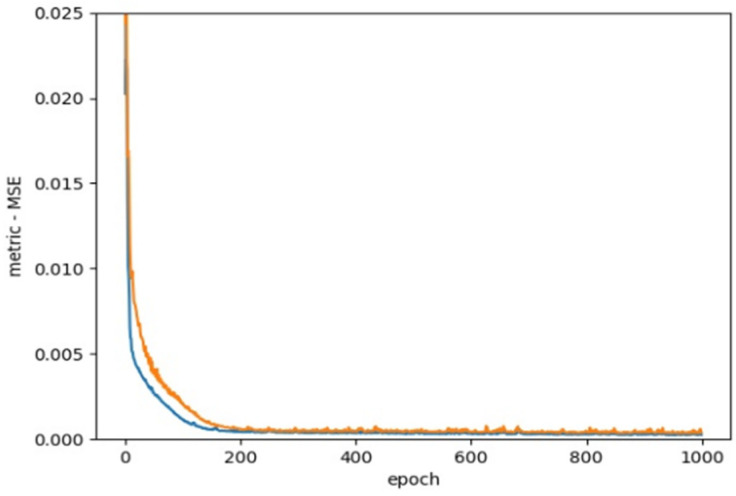
Loss function, MSE.

As Figure 4 presents, we use MSE as the loss function based on previous studies.

One of the essential principles in engineering research is the stochastic gradient-based optimization of parametric functions to maximize or minimize function parameter values. Adaptive moment estimation (ADAM) is an efficient method in deep learning that can be applied for first-order gradient-based optimization of stochastic objective functions by using momentum and adaptive learning rates to accelerate convergence. ADAM can work with sparse gradients, and the significance of parameter updates is constant to gradient rescaling.

Empirical results have shown that ADAM’s performance is helpful in practice and better than other random optimization methods, such as AdaGrad, RMSProp, AdaDelta, and SGDNesterov SFO (sum-of-functions optimizer) [66,67,68,69,70,71]. In this research, we used the ADAM algorithm to optimize the model.

### 3.3. Model Evaluation Method

After obtaining the required multilinear regression equation, we needed to judge the regression equation’s goodness of fit, carried out through model evaluation [72].

For multilinear regression, the most commonly used model evaluation indicator is likely the mean square error (MSE):

(7) $$\mathrm{MSE}=\frac{1}{n}\overset{n}{\underset{i=1}{\sum }}{\left(y_{i}-\hat{f}\left(x_{i}\right)\right)}^{2}$$

This is the prediction at the ith observation point. Suppose the response value of the predicted value is very close to the actual response value. In that case, the MSE is very small. In contrast, if there is a material difference between the predicted response value and the actual response value, the MSE is very large. When the MSE is calculated from training data, it is called training MSE. However, our general relationship is calculated for the test data (i.e., test error means square error). The appropriate model must be selected to minimize the test square error.

For multilinear regression, another commonly used model evaluation indicator is the multiple determination coefficient (multiple coefficients of determination; $R^{2}$). The multiple determination coefficient is a statistic that measures the fit of multiple regression equations. It reflects the proportions explained by the estimated regression equations in the variance of the factor variable y, calculated as the progression squares’ proportion to the sum of total squares. The greater the goodness of fit, the higher the degree to which the argument interprets the cause variable. The higher the percentage of change caused by the argument to the total transformation, the denser the observation points are near the regression line.

(8) $$R^{2}-\frac{SSR}{SST}=1-\frac{SSE}{SST}$$

where SST (total sum of squares) is the sum of squares, SSR (regression sum of squares) is the sum of regression squares, and SSE (error sum of squares) is the sum of residual squares.

(9) $$SSR=\overset{m}{\underset{i=1}{\sum }}{\left({\hat{y}}_{i}-\bar{y}\right)}^{2}$$

(10) $$SSE=\overset{m}{\underset{i=1}{\sum }}{\left(y_{i}-\bar{y}\right)}^{2}$$

where ${\hat{y}}_{i}$ represents the model forecast value and $\bar{y}$ indicates the average of y.

### 3.4. Comparison of ABAQUS and ANN Results

**Figure 5:**
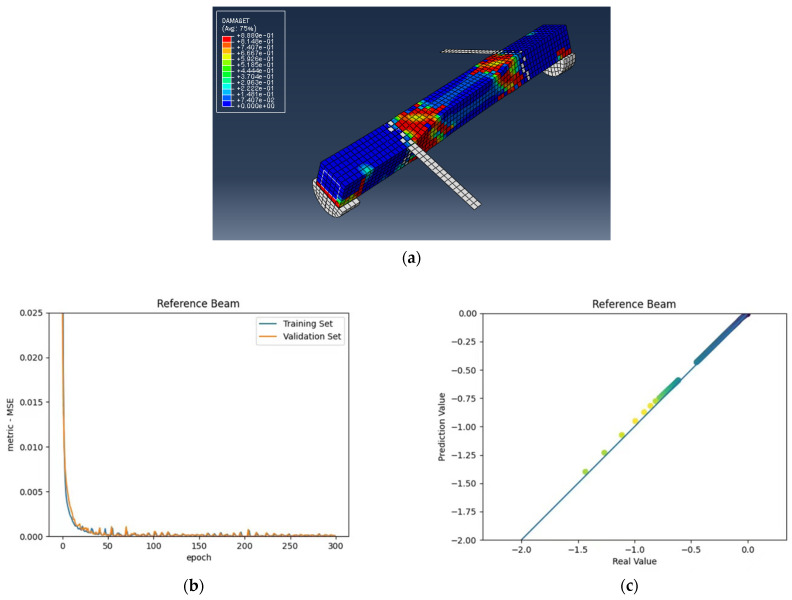
(**a**) Reference beam under combined torsion and bending, (**b**) Training loss, (**c**) Accuracy.

**Figure 6:**
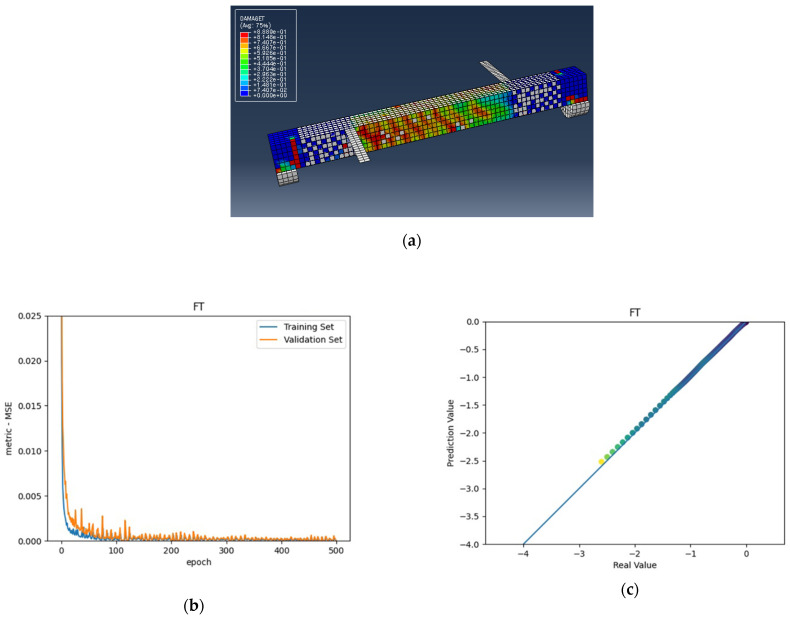
(**a**) FT beam under combined torsion and bending, (**b**) Training loss, (**c**) Accuracy.

**Figure 7:**
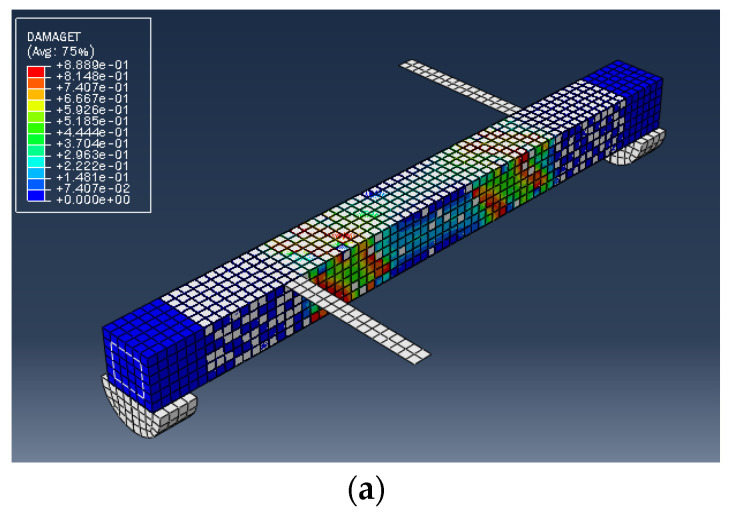

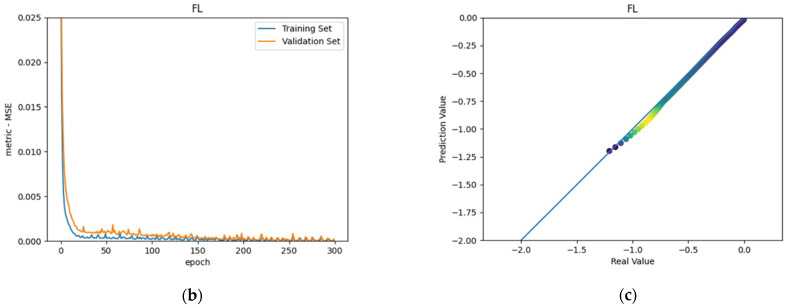
(**a**) FL beam under combined torsion and bending, (**b**) Training loss, (**c**) Accuracy.

**Figure 8:**
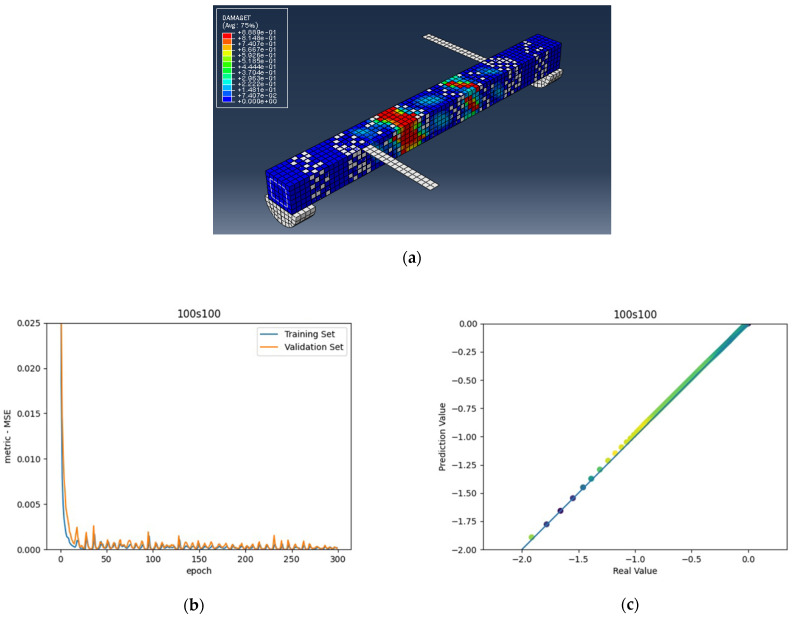
(**a**) 100s100 beam under combined torsion and bending, (**b**) Training loss, (**c**) Accuracy.

**Figure 9:**
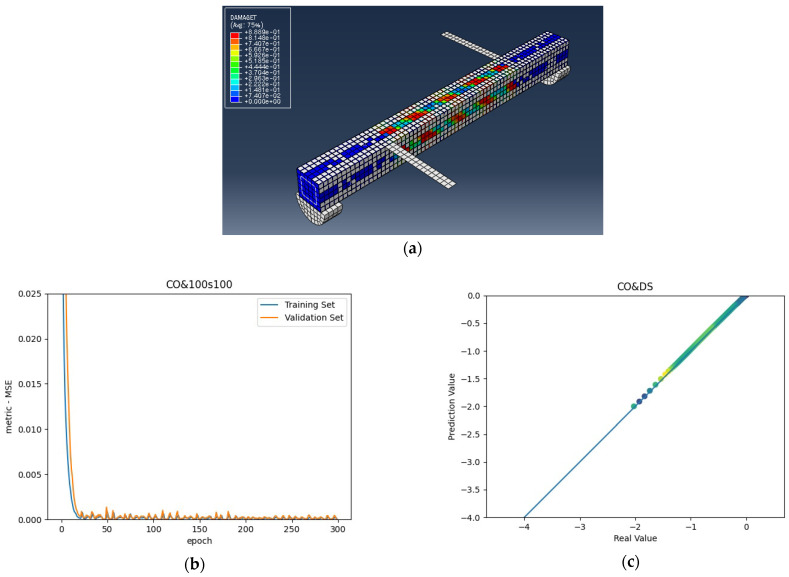
(**a**) CO and 100s100 beam under combined torsion and bending, (**b**) Training loss, (**c**) Accuracy.

**Figure 10:**
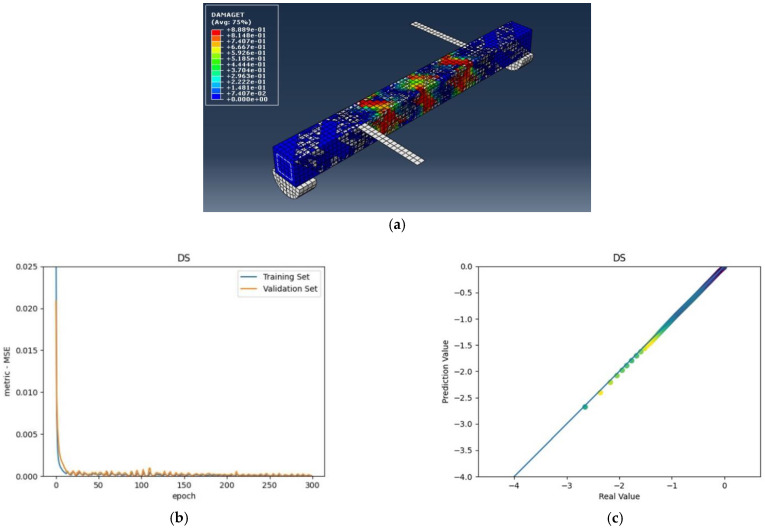
(**a**) DS beam under combined torsion and bending, (**b**) Training loss, (**c**) Accuracy.

**Figure 11:**
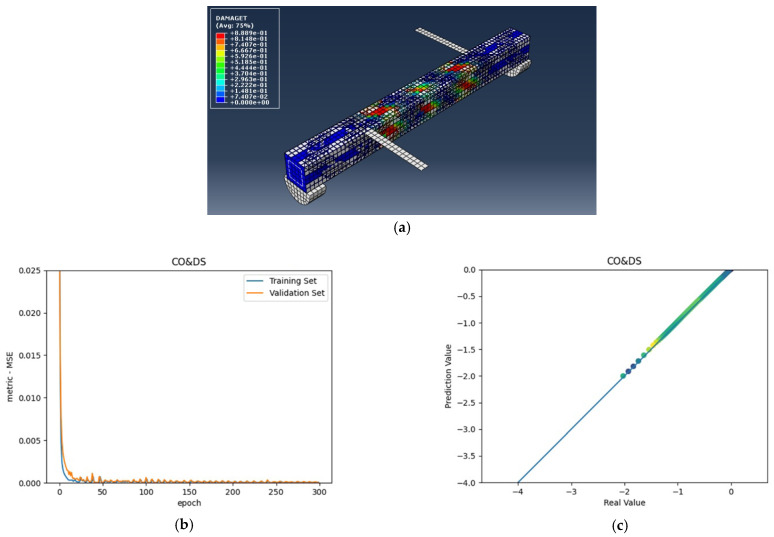
(**a**) CO and DS beam under combined torsion and bending, (**b**) Training loss, (**c**) Accuracy.

The numerical analysis of all beams above confirms that using GFRP strips as external retrofitting has desirable effects and performances in increasing the ultimate strength and significantly decreasing the crack growth, as shown in the following figures. Figure 5, Figure 6 and Figure 7 present the numerical modeling and ANN results of the reference, FT, and FL beams under combined torsion and bending. Moreover, Figure 8, Figure 9, Figure 10 and Figure 11 show the ABAQUS modeling and ANN results of 100s100, CO and 100s100, DS, and CO and DS beams, respectively. Scatter plots of target values versus predicted ANN values are shown in this section. MSE performs as the loss function during data training by ANN.

As shown in the above pictures, after a limited number of ANN iterations, MSE can obtain a sufficiently small value on the training set and validation set. Meanwhile, the predicted value can perfectly fit the actual value, as seen from the scatter diagram. In the subfigures (c) of Figure 5, Figure 6, Figure 7, Figure 8, Figure 9, Figure 10, and Figure 11, the line indicates where the predicted value is equal to the actual value. The closer the scatter distance is to the line, the more accurate the predicted value is.

We can see that the ANN perfectly fits the data. As Table 7 shows, from the MSE point of view, we can observe that for reference, FT, FL, 100s100, CO and 100s100, DS, and CO and DS beams with MSEs of 0.000371, 0.000758, 0.000769, 0.000324, 0.000167, 0.000504, and 0.000591 in the training set, respectively, all have MSE values in the training set so small that they are lower than 0.0008. Furthermore, with MSEs of 0.000404, 0.000812, 0.000988, 0.000380, 0.000169, 0.000689, and 0.000619 in the test set, all MSE values in the test set are also small and lower than 0.000999.

Considering the $R^{2}$ values, we can observe that the reference, FT, FL, 100s100, CO and 100s100, DS, and CO and DS beams have $R^{2}$ values of 0. 996172, 0.998041, 0.991427, 0.998172, 0.999827, 0.998447 and 0.997394 in the training set, respectively. All $R^{2}$ values in the training set are so close to 1 that they are more significant than 0.980000. In addition, their $R^{2}$ values in the test set are 0.993720, 0.997069, 0.984757, 0.996783, 0.999781, 0.996769 and 0.996143, respectively. All $R^{2}$ values in the test set are also close to 1, and they are greater than 0.980000. This has shown that the ANN perfectly fits the data.

## 4. Conclusions

This study presented finite element analysis using ABAQUS to investigate the strength increase in retrofitted and non-retrofitted reinforced concrete beams with different FRP orientations. The dimensions, end supports, and specifications of the beam models were identical to those of the experimental studies. Artificial neural networks helped us predict the structural behavior results and prove our research work’s accuracy. The final comparison of numerical analysis and experimental results yielded the following significant results:

1. As presented in the figures and tables, the numerical results of the structural behavior are desirably consistent with the experimental results of all the beams.
2. Similar to experimental results, numerical results showed that FRP materials increase the ultimate torsional strength and the angle of twist in retrofitted versus non-retrofitted beams. In most cases, the ultimate torque and proper angle of twist obtained from numerical analysis are conservatively lower than the experimental study’s corresponding values.
3. Numerical solutions have shown that fiber orientation substantially influences the ultimate torsional strength and proper ultimate angle of the twist.
4. Based on the numerical results, it can be concluded that the strength of retrofitted beams depends on the volumetric ratio and type of orientation of the employed FRP reinforcement. For a certain twist angle, beams with higher torsional reinforcement have a greater torsional capacity, increased post-cracking stiffness, and ultimate angle of twist.
5. According to the MSE and $R^{2}$ results, the ANN could accurately predict the experimental and ABAQUS results. The data prove the precision of the ANN prediction results for RC beam structural behavior retrofitted with FRP sheets.

## Figures and Tables

**Table 1:** Diverse wrapping configurations used for retrofitting.

Beam	Wrapping Configurations
Control Beam	N/A
FT	Full Transverse Wrapping
FL	Full Longitudinal Wrapping
100S100	100 mm Strip Wrapping at 100 mm c/c
CO and 100S100	Corner and 100 mm Strip Wrapping at 100 mm c/c
DS	Diagonal Strip Wrapping
CO and DS	Corner and Diagonal Strip Wrapping

**Table 2:** Material properties of GFRP.

FRP	$E\;\left(N/mm^{2}\right)$	$\mathrm{Tensile}\;\mathrm{Strength}\;\left(N/mm^{2}\right)$	Thickness (mm)
GFRP	74,500	3400	0.324

**Table 3:** Summary of beam tests.

Designation	Combined Effects	Mean Concrete Strength (MPa)	FRP	Bending Moment at First Crack (KN·m)	The Torsional Moment at First Crack (KN·m)	Maximum Torsional Resistance (KN·m)
BTCON	Torsion and Bending	25	-	2.279	3.631	3.8
FT	Torsion and Bending	25	GFRP	5.279	8.331	8.4
FL	Torsion and Bending	25	GFRP	3.516	5.57	5.7
100S100	Torsion and Bending	25	GFRP	2.841	4.512	5.8
CO & 100S100	Torsion and Bending	25	GFRP	4.041	6.392	7.2
DS	Torsion and Bending	25	GFRP	4.041	6.392	8.2
CO&DS	Torsion and Bending	25	GFRP	4.829	7.626	8.7

**Table 4:** Cracking torque, ultimate torque, ultimate angle of twist, and effects of fibers on the cracking torque, ultimate torque, ultimate angle of twist for FT, FL, 100s100, CO and 100s100, DS, and CO and DS (experimental study).

Beam	$T_{cr}\;$ (KN·m)	$T_{u}\;$ (KN·m)	Ultimate Angel of $Twist\;\emptyset_{u}\;$ (Deg/m)	$T_{cr\left(GFRP\right)}\;\left(KN\cdot m\right)\;$	$T_{u\left(GFRP\right)}\;\left(KN\cdot m\right)\;$	$\emptyset_{u\left(GFRP\right)}\;$ (Deg/m)
Reference Beam	2.7	4.1	5.8	-	-	-
FT	6.8	8.4	11.6	4.1	4.3	5.8
FL	4.9	4.9	4.9	2.2	0.8	-
100S100	3.95	6	8.05	1.25	1.9	2.25
CO and 100S100	6	7.15	17.5	3.3	3.05	11.7
DS	6.9	8.65	11.1	4.2	4.55	5.3
CO and DS	5.9	7.9	8.9	3.2	3.8	3.1

**Table 5:** Cracking torque, ultimate torque, ultimate angle of twist, and effects of fibers on the cracking torque, ultimate torque, ultimate angle of twist for FT, FL, 100s100, CO and 100s100, DS, and CO and DS (Numerical analysis).

Beam	$T_{cr}\;$ (KN·m)	$T_{u}\;$ (KN·m)	Ultimate Angel $of\;Twist\;\emptyset_{u}\;$ (Deg/m)	$T_{cr\left(GFRP\right)}\;$ (KN·m)	$T_{u\left(GFRP\right)}\;$ (KN·m)	$\emptyset_{u\left(GFRP\right)}\;$ (Deg/m)
Reference Beam	3.2	3.97	6	-	-	-
FT	7.1	8.46	11.048	3.9	4.49	5.048
FL	4.8	5.076	5.14	1.6	1.106	-
100S100	5.2	6.016	8.14	2	2.046	2.14
CO and 100S100	5.5	7.23	17.37	2.3	3.26	11.37
DS	7.7	8.64	11.27	4.5	4.49	5.27
CO and DS	7.1	7.91	8.61	3.9	3.94	2.61

**Table 6:** Comparison of the numerical and experimental values of the cracking torque, ultimate torque, and ultimate angle of the twist, GFRP.

Beam	$T_{crN}\;$ (KN·m)	$T_{uN}\;$ (KN·m)	$\emptyset_{uN}\;$ (Deg/m)	$T_{crE}\;$ (KN·m)	$T_{uE}\;$ (KN·m)	$\emptyset_{uE}\;$ (Deg/m)	$\frac{T_{crN}\;}{T_{crE}}\;$	$\frac{T_{uN}}{T_{uE}}\;$	$\frac{\emptyset_{uN}}{\emptyset_{uE}}\;$
Reference Beam	-	-	-	-	-	-	-	-	-
FT	3.9	4.49	5.048	4.1	4.3	5.8	0.95	1.044	0.87
FL	1.6	1.106	-	2.2	0.8	-	0.72	1.38	-
100S100	2	2.046	2.14	1.25	1.9	2.25	1.6	1.07	0.95
CO and 100S100	2.3	3.26	11.37	3.3	3.05	11.7	0.7	1.07	0.97
DS	4.5	4.49	5.27	4.2	4.55	5.3	1.07	0.98	0.99
CO and DS	3.9	3.94	2.61	3.2	3.8	3.1	1.21	1.036	0.84

**Table 7:** Evaluation of statistical parameters of the ANN model.

Beam Model	Evaluation Index					
	Training Set		Test Set		All Set	
	MSE	$R^{2}$	MSE	$R^{2}$	MSE	$R^{2}$
Beam	0.000371	0.996172	0.000404	0.99372	0.000377	0.995841
FT	0.000758	0.998041	0.000812	0.997069	0.000769	0.997908
FL	0.000769	0.991427	0.000988	0.984757	0.000812	0.990513
100s100	0.000324	0.998172	0.00038	0.996783	0.000335	0.997988
CO and 100s100	0.000167	0.999827	0.000169	0.999781	0.000167	0.99982
DS	0.000504	0.998447	0.000689	0.996769	0.000541	0.998225
CO and DS	0.000591	0.997394	0.000619	0.996143	0.000596	0.997234
